# Prevalence and associated occupational factors of low back pain among the bank employees in Dhaka City

**DOI:** 10.1002/1348-9585.12131

**Published:** 2020-07-27

**Authors:** Mohammad Ali, Gias U. Ahsan, Ahmed Hossain

**Affiliations:** ^1^ Department of Physiotherapy Uttara Adhunik Medical College Hospital Dhaka Bangladesh; ^2^ Centre for Higher Studies and Research Bangladesh University of Professionals Dhaka Bangladesh; ^3^ Department of Public Health North South University Dhaka Bangladesh; ^4^ Health Management BD Foundation Dhaka Bangladesh

**Keywords:** Bangladesh, bank employees, low back pain, occupational health, random forest

## Abstract

**Objective:**

Low back pain (LBP) is one of the common health problems among full‐time office employees that causes absenteeism from work. The aim of the study is to identify the association between occupational factors and LBP among full‐time bank employees in Dhaka City.

**Materials and Methods:**

We conducted a cross‐sectional study involving 593 full‐time bank employees who were engaged in sedentary works. The 1‐month complaint of LBP was measured using a subscale of subjective health complaints inventory. Multivariable logistic models were performed to identify variables related to LBP, and a random forest technique was performed to determine the top five important variables.

**Results:**

The 1‐month prevalence for LBP was found to be 36.6% among the bank employees, and the prevalence was the highest (64.3%) for the 51‐ to 59‐year‐old age group. The regression analysis indicates that respondents from both agegroups, 41‐50 years (OR = 2.00, 95% confidence interval [CI] = 1.10‐3.69) and 51‐59 years age groups (OR = 5.14, 95% CI = 2.05‐13.64), are significantly associated with LBP. Furthermore, obesity (OR = 2.06, 95% CI = 1.01‐4.21), and prolong working hours (>9 hours) (OR = 1.42, 95% CI = 1.01‐2.0) are positively associated with LBP. The top five important variables for LBP identified by random forest technique are: age, length of employment, prolong office hours, presence of chronic illness, and physical activity.

**Conclusion:**

LBP is highly prevalent in full‐time bank employees. The occupational factors, including the length of employment (>10 years) and long working hours, play a significant role in developing LBP among bank employees. Moreover, several factors, including age, chronic illness, obesity, and physical activity, should be taken into account in the prevention of LBP in bank employees.

## 1 BACKGROUND

The global burden of diseases, injuries, and risk factors study in 2016 suggested that among 328 morbidities, low back pain (LBP) became one of the primary health concerns for any population group.^1^ LBP can induce an absence of enthusiasm, mental unrest, and physical discomfort or burden on its bearer.^2^ Consequently, LBP became a significant cause of taking sick leave and early retirement among the working population.^3^

Globally, several studies have reported a 1‐month LBP prevalence among office workers ranging from 23% to 46%.^4^, ^5^, ^6^, ^7^ The prevalence of LBP has been found higher in low‐income countries compared with high‐income countries.^4^, ^8^ In addition, the prevalence of employment‐related LBP in Bangladesh was found to be high in different job settings.^9^, ^10^, ^11^ A study of female nurses in Bangladesh, for example, found that around 31% of nurses had chronic LBP.^10^ Another research among Bangladesh's garment workers reported that chronic LBP prevalence was 38.60%.^11^ There is still a research discrepancy in recognizing the prevalence of full‐time workers engaged in sedentary jobs.

Occupation‐related factors are inextricably associated with LBP. It is estimated that 37% of LBP is due to risk factors at work around the world.^12^ Bank employees usually spend a long proportion of their sedentary time sitting during working hours, which is associated with an increased risk of various long‐term health conditions. A study revealed that long‐time sedentary work, high workload, and inappropriate sitting arrangements are the contemporary causes of LBP.^13^ Several studies with office staff have established a relationship between sitting and LBP.^14^, ^15^, ^16^, ^17^, ^18^, ^19^ A study suggested that the risk factors of LBP for office workers were: long office hours, working in the same posture, and continuing the same job for many years.^2^ Furthermore, few studies revealed that prolonged sitting was associated with metabolic disorders, sleep disturbance, hypertension, and high body mass index (BMI).^20^, ^21^ These factors are also positively associated with increased LBP.^22^

Despite many articles published in identifying the associated factors of LBP among office workers, there is still a limited understanding of the occupational factors that influence LBP in sedentary workers of Bangladesh. Thus, this study aims to determine the prevalence of LBP and find the associated occupational factors among bank employees in Dhaka City.

## 2 METHODOLOGY

### 2.1 Study site and study population

We conducted an analytical cross‐sectional study in Dhaka City between December 2018 and May 2019. Dhaka is the capital city and economic hub of Bangladesh. There are 50 banks in Dhaka City, and we conveniently selected 32 banks to collect data from their full‐time employees. Participants who met the following criteria were eligible for the enrollment as study participants:

#### 2.1.1 Inclusion criteria

1. We selected full‐time bank employees who maintained a regular office hour for at least 1 year in banks.
2. Age: 18‐59 years old.
3. The bank employees, who worked mainly in a sitting environment.
4. Those who were willing to participate in the study.

#### 2.1.2 Exclusion criteria

1. Pregnant women or female employees who have a baby younger than 6 months.
2. Previous surgical history (surgery in the pelvic region, spinal surgery, LUCS, etc).
3. Any history of back injury or any other spinal injury caused by accident (eg, road accident, etc).
4. Any history of joint or bone disorder or prolapse lumbar intervertebral disc.
5. Any history of chronic inflammatory pain (eg, rheumatoid arthritis, ankylosing spondylitis, etc).

### 2.2 Sampling technique and sample size

A convenient sampling technique was used to select the bank employees from banks following the STROBE guideline (https://www.strobe‐ statement.org/index.php?id = available‐checklists).

The minimum necessary sample size for the study was calculated based on a 95% confidence interval (CI) and assuming the prevalence of LBP among full‐time employees as 35%. We calculated the minimum required sample as 546 by considering a 4% marginal error.

**Figure 1:**
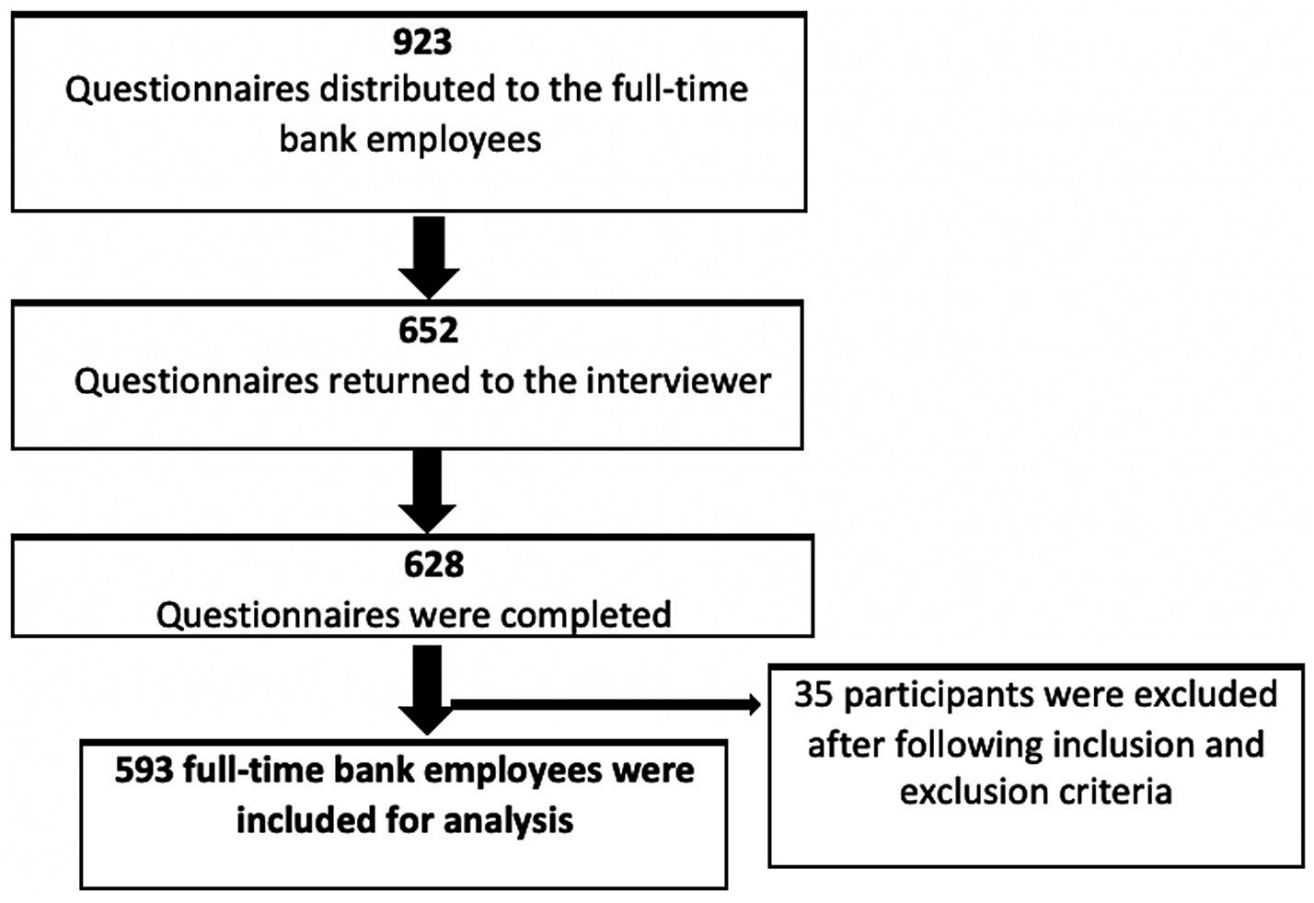
Flow chart of the data collection

Figure 1 represents the flow chart of the data collection. We distributed 923 paper‐based questionnaires to the employees during office hours. During the study period, we collected 652 questionnaires of which 628 were completed. After subsequent elimination based on inclusion and exclusion criteria, we included 593 participants in our analysis.

### 2.3 Dependent variable

The questions on LBP were based on the musculoskeletal subscale of subjective health complaints produced by Eriksen et al that measures LBP complaints experienced in the last month.^23^ Employees were asked to rate the occurrence of pain or discomfort in the lower back with four answering categories (“no complaint,” “only once/a little,” “of short duration/some,” “frequently/ serious”). Employees who answered, “no complaint” or “only once/a little” on LBP were classified as having no LBP. Those who answered “of short duration/some” or “frequently/serious” were classified as having complaints of LBP.

### 2.4 Independent variables

Data on sociodemographic factors, age, gender, BMI (calculated based on weight and height), and marital status, were collected using a semi‐structured questionnaire. Behavioral factors including sleep arrangements (firm or foam mattresses), smoking habits (current, previous, or never), and physical activities of the respondents were collected. The response of sleep arrangement by a firm or foam mattress was subjective about the feel of rigidness about the mattress. Physical activities were calculated based on the metabolic equivalents (MET minutes/week) scale. In this study, the levels of physical activity of the respondents were measured by asking about their weekly activities during work and leisure time, activities related to transport, and time spent in a sedentary position. MET minute was calculated according to the STEPS protocol, and physical activity was categorized into moderate to vigorous, light, and sedentary activity.^24^ We also collected data on occupational factors, including the length of employment and average daily working hours. Crowding was calculated by dividing the number of family members in the house by the number of bedrooms. Data on common chronic illness (diabetes and hypertension) from the employees were also collected.

### 2.5 Statistical analysis

We analyzed the data using software R. We presented the categorical variables as frequencies and percentages in the two LBP categories. Chi‐squared tests were used to compare categorical variables in employees with and without LBP. We investigated the association between LBP and exposure variables using a multivariable logistic regression model and calculated adjusted OR (AOR) for each factor. The results were reported by odds ratios (ORs) and corresponded 95% confidence intervals (CIs). P‐values less than 0.05 were considered statistically significant. We also used the random forest (RF) method that allows for nonlinear relationships between independent variables. The machine learning technique can allow the correlated variables to identify robust predictors, which are defined as important variables. We used the function randomForest() from the package of randomForest in R with a training dataset of 70% of the complete dataset.

## 3 RESULT

### 3.1 Univariate Analysis

Among the 593 respondents, there were 342 (57.7%) males and 251 (42.3%) females. The descriptive statistics of the sociodemographic factors, such as age, gender, BMI, marital status, crowding, and sleeping arrangements, are described in Table 1. Nearly three‐fifths (59.02%) of the participants were between the ages of 31 and 40 and about half of the workers were either overweight or obese (50.3%).

**Table 1:** Univariate analysis: Sociodemographic factors and LBP

Factors	Categories	Low back pain (LBP)		Total (%) within categories	*P*‐value*
		No (Row %)	Yes (Row %)		
Age (Years)	≤30	85 (71.2)	32 (28.2)	117 (19.7%)	**<.001**
	31‐40	227 (64.9)	123 (35.1)	350 (59.1%)	
	41‐50	55 (56.1)	43 (43.9)	98 (16.5%)	
	51‐59	9 (35.3)	19 (64.3)	28 (4.7%)	
Gender	Female	154 (61.3)	97 (38.7)	251 (42.3%)	.422
	Male	222 (64.9)	120 (35.1)	342 (57.7%)	
BMI	Healthy weight	199 (67)	95 (32.9)	295 (49.7%)	**.002**
	Overweight	162 (61.9)	99 (38.1)	260 (43.8%)	
	Obese	15 (44.7)	23 (55.3)	38 (6.5%)	
Marital Status	Married	304 (61.2)	193 (38.8)	497 (83.8%)	**.014**
	Unmarried	72 (75.0)	24 (25.0)	96 (16.2%)	
Crowding	≤1.5	202 (64.0)	115 (36.0)	317 (53.5%)	.833
	1.5‐2.0	117 (62.4)	72 (37.6)	189 (31.9%)	
	2+	57 (63.2)	30 (36.8)	87 (14.6%)	
Sleeping arrangement	Firm mattress	316 (62.9)	186 (37.1)	502 (84.7%)	.670
	Foam mattress	60 (65.3)	31 (34.7)	91 (15.3%)	

Bold values represent significant at 5% significance level.

* *P*‐value is calculated from chi‐squared test.

**Figure 2:**
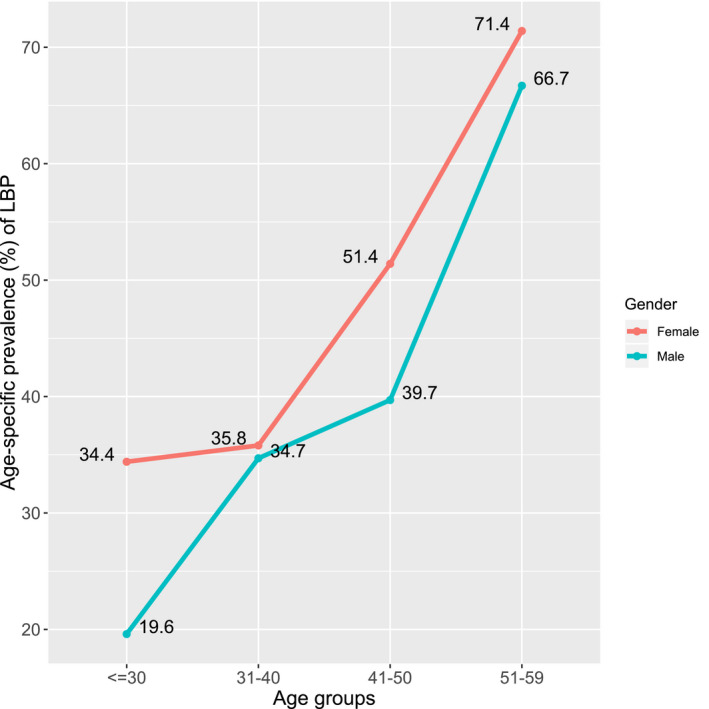
Age‐specific prevalence of LBP by gender

The 1‐month prevalence of the complaints about LBP is found to be 36.6% among the bank employees. A line diagram is shown in Figure 2 to understand the relationship between age‐specific LBP by gender. The figure shows that females are more prone to chronic LBP than males, whatever their age. Through age, the prevalence of LBP appears to have increased, and is more prevalent among female workers. The figure also reveals that the highest prevalence (71.4%) of LBP was among female workers in the 51‐ to 59‐year‐old age group, indicating that it is a common condition among older women. Tests of the chi‐squared statistics *P*‐value from Table 1 indicate that age has a major correlation with LBP. Most of the participants were married in this study (83.8%), and they appeared to complain more about LBP compared to unmarried participants. More aged workers have been married though. And so, in the multivariable logistic regression model, we omitted marital status in order to prevent collinearity.

The behavioral and occupational factors of the participants, such as smoking habits, chronic illness, physical activity, length of the employment in a bank (years), and average working hours per day, are described in Table 2. Nevertheless, those who extended prolonged working hours (more than 9 hours in the workplace/day) complained more about LBP than the workers who held daily regular office hours (41.5% and 32.7%, respectively). With the growing length of employment in banks, a gradual pattern of complaints about LBP among the employees was observed. In a bank, the correlation between age and length of employment was found to be 0.87, suggesting a high correlation. In the multivariable logistic regression analysis, we included age and omitted length of employment in order to prevent collinearity within the model. In addition, 46.6% of employees with any chronic condition (diabetes or hypertension) reported LBP (*P* = .009).

**Table 2:** Univariate analysis: Behavioral and occupational factors on LBP

Factors	Categories	Low back pain (LBP)		Total (%) within categories	*P*‐value*
Smoking habit		No (Row %)	Yes (Row %)		.740
	Never	310 (63.0)	182 (37.0)	492 (83.0%)	
	Current/ Previous	66 (65.3)	35 (34.7)	101 (17.0%)	
Chronic illness	No	306 (66.2)	156 (33.8)	462 (77.9%)	**.009**
	Yes	70 (55.4)	61 (46.6)	131 (22.1%)	
Physical Activity	Sedentary	110 (60.5)	78 (41.5)	188 (31.7%)	.216
	Light	231 (65.3)	123 (34.7)	354 (59.7%)	
	Moderate‐Vigorous	35 (68.6)	16 (31.4)	51 (08.6%)	
Length of employment (y)	≤5	141 (73.4)	51 (26.6)	192 (32.4%)	**<.001**
	6‐10	127 (63.3)	72 (36.7)	199 (33.6%)	
	10+	108 (54.0)	94 (46.0)	202 (34.0%)	
Working hours per day	Regular (8‐9)	224 (66.3)	109 (32.7)	333 (56.2%)	**.033**
	Long (>9)	152 (58.5)	108 (41.5)	260 (43.8%)	

Bold values represent significant at 5% significance level.

* *P*‐value is calculated from chi‐squared test.

### 3.2 Multivariable logistic regression model

We fit a multivariable logistic regression model with the complaints of LBP after adjusting the variables contained in Tables 1 and 2 with *P‐*values less than 0.5. *P*‐values were calculated in multivariable logistic regression for each variable, and were considered statistically significant at the level of significance .05. It appears from Table 3 that there are four factors associated with the complaints of LBP at a 5% significance level. The four variables were found to have a significant association with LBP: prolonged working hours a day (OR = 1.45), older age groups (41‐50 years, OR = 2.0; 51‐59 years, OR = 5.14), BMI category obese (OR = 2.65), and light physical activity (OR = 0.67). The findings show that the bank workers who work long hours are 1.45 times more likely than those who work normal hours to encounter LBP. Additionally, the odds of getting LBP for workers aged between 51 and 59 years are 5.14 times higher compared to the age group under 30 years. Obesity (OR = 2.65) is found to be significant, and the odds ratio shows that an obese person is 2.65 times more likely to have LBP than a healthy weight group person.

**Table 3:** Result from multivariable logistic regression model

Factors	Categories	AOR (95%CI)	*P*‐value
Age (y)	≤30	Reference	
	31‐40	1.45 (0.91‐2.36)	.126
	41‐50	2.00 (1.10‐3.69)	.024**
	51‐59	5.14 (2.05‐13.64)	.001**
Gender	Male	Reference	.146
	Female	1.33 (0.91‐1.95)	
BMI	Healthy weight	Reference	
	Overweight	1.21 (0.84‐1.74)	.314
	Obese	2.65 (1.30‐5.55)	.008**
Office hours	Regular (8‐9 h)	Reference	0.038**
	Long (>9 h)	1.45 (1.02‐2.06)	
Chronic diseases (diabetes or cardiovascular)	Yes	1.35 (0.86‐2.09)	.178
	No	Reference	
Physical activity	Sedentary	Reference	
	Light	0.67 (0.46‐0.97)	.036**
	Moderate to vigorous	0.52 (0.25‐1.03)	.070

** Significant at 5% significance level.

### 3.3 Variables with high importance in random forest model

**Figure 3:**
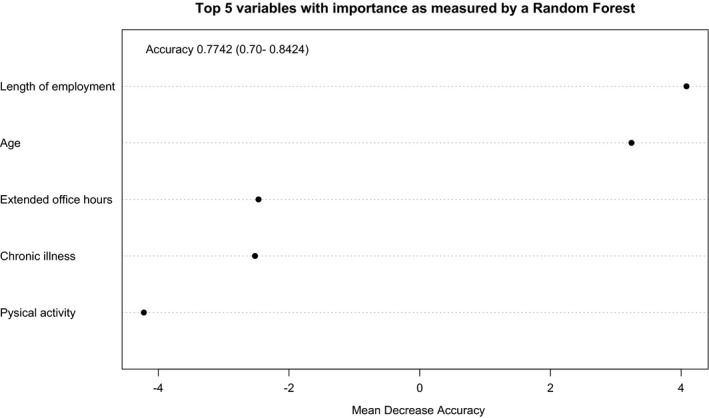
Top five importance of variables as measured by a random forest

Here we view the importance of variables by applying a random forest model when predicting LBP. There is agreement that "overfitting" rarely affects random forests, which plagues many other models.^25^ We used mean decrease accuracy to assess random forests and to examine them on the probability of affecting LBP. Figure 3 shows the outcome of the random forest model, which indicates a mean decrease in accuracy for the variables. The overall accuracy of test data is 77.42%. The most important variables in the top 3 tend to be: length of employment, age, and long working hours. Occupational factors therefore play a major role in LBP complaints.

### 3.4 Worldwide period‐prevalence comparison

Low back pain is one of the most common chronic illnesses in the world. A number of publications have documented LBP prevalence and risk factors. However, most studies available compare various database types, LBP definitions, dates, etc, restricting the accuracy of such intercountry comparisons to a significant degree. The 1‐month LBP prevalence among bank employees was found to be 36.6% in our study.

The prevalence of LBP from 10 selected studies is listed in Table 4. Prevalence of LBP in the past month among office employees in Costa Rica, Nicaragua, and Spain was 46%, 44%, and 33.6%, respectively.^4^ One‐month prevalence of LBP among employees of school for handicapped children in Japan was 45%.^5^ Moreover, a systematic analysis conducted in 2012 indicated a global prevalence of LBP of 23.2 ± 2.9% for 1 month.^6^ Additionally, 1‐month prevalence of LBP among different manual workers and the general population in the USA was 39% and 44%, respectively.^7^ Among Thai university office workers, estimated 3‐month prevalence of LBP was 52.8%.^26^ Similar to our findings, a cross‐sectional analysis carried out in Kigali, Rwanda found that 45.8% of bank employees encountered LBP in 1 year.^27^ In addition, studies conducted among office employees in Nigeria and Malaysia found a prevalence of 38% and 37% in 12 months, respectively.^28^, ^29^ Lifetime prevalence of LBP was estimated to be 69.2% among Pakistani office workers.^30^ In India, the 1‐year prevalence of LBP among IT professionals was 51%.^31^ On the other hand, the 12‐month prevalence of LBP in Australian adults was 67%.^32^ Global point prevalence, 1‐month prevalence, and 12‐months prevalence of LBP among the adult population were estimated at 12%, 23%, and 38%, respectively.^33^ Therefore, the 1‐month prevalence of LBP ranged from 23% to 46%, and it depends on the form of questionnaire used, the length of the target population's prevalence, occupational provision, and lifestyle, along with the economic situation of the country where the study was carried out.

**Table 4:** Prevalence of LBP in countries from 10 selected published articles

Country and source	Period‐prevalence	Population	Prevalence
Costa Rica, Nicaragua, and Spain ^4^	1 month	Three Spanish speaking country, office workers	46%, 44%, and 33.6%, respectively
Japan ^5^	1 month	School staffs	45%
Global prevalence ^6^	1 month	General population	23.2 ± 2.9%
USA^7^	1 month	Manual workers and General adult population	39% and 44%, respectively
Kigali, Rwanda^27^	12 months	Bankers	45%
Nigeria^28^	12 months	Office workers	38%
Pakistan^30^	Lifetime	Office workers	69.2%
India^31^	12 months	Information technology professionals	51%
Malaysia^29^	12 months	Office workers	37%
Thailand^26^	3 months	Office workers	52.8%

## 4 DISCUSSION

Around one‐third of bank employees reported LBP in this survey. In fact, this is consistent with other population studies from developing countries.^4^, ^5^, ^6^, ^7^ The findings of this study suggest that among bank employees, a long office hour and a growing year of employment were independently correlated with LBP. A study carried out in Denmark also indicated that the workers who spent a long time at the office reported LBP at a higher rate than those who worked less in a sitting environment.^34^ A similar finding was published in another study conducted with university employees in Qatar.^2^ The bank workers who had served for more than 10 years complained more about LBP than those who had served for less than 5 years. Furthermore, an increased prevalence of LBP has been identified for office staff with a rising year of employment.^21^, ^34^, ^35^

Increasing age is a significant risk factor in developing LBP.^36^ Our findings indicate that older adults, 50 years of age or older, reported LBP more frequently than young adults. Although the majority of LBP triggers in older adults are nonspecific and self‐limiting, senior bank employees are vulnerable to developing such LBP due to their age‐related physical and psychosocial changes. Sadly, numerous factors that may affect successful LBP management among older adults have not been discussed by us. Accordingly, the aims of the current article were to comprehensively summarize common and related factors of developing LBP in adults, to highlight particular problems in evaluating and treating full‐time workers with LBP, and to explore possible directions for study. In this regard, we may propose that senior bank workers may use work from home strategy, which is more common in developed countries. This work from home strategy may reduce the issue of commuting to office and preventing traffic congestion. Unfortunately, in our study, we did not include these factors and so we cannot comment on such strategies.

Therefore, by jointly understanding the effects of various factors on the evaluation and treatment of older adults with LBP, both physicians and researchers should work toward a more cost‐effective and personalized guideline for older people.

A positive association was observed between the variables of marital status and LBP. Similar findings were also found in a survey of Iranian population.^37^ A significant point to remember is that we found the majority of older workers to be married in our sample and so the variable may play a confounding role in the association.

We found that there is a clear positive correlation between obesity and LBP complaints. A meta‐analysis has suggested that obesity has been associated with increased LBP prevalence over the past 12 months.^38^ In our sample, female workers complained more about LBP but in the multivariable logistic analysis, gender was not found significant at 5% significance level. The odds ratio shows that, relative to male workers, female employees complained LBP 33% more. A systematic review also indicated that the prevalence of LBP was increased for women relative to men.^39^ There were also few studies that indicated routine physical activity could reduce LBP.^9^, ^40^ From our analysis, it appeared that bank workers had an insufficient degree of physical activity practice. The multivariable results indicate a substantial correlation between physical activity and LBP, and the factor was also established as an essential variable by the random forest method. This indicates that a lighter level of physical activity over a longer period of time can influence the reduction of LBP complaints. Further intervention studies are required to clarify the role that physical activity plays in LBP prevention.

### 4.1 Limitations

The key limitation of this study (as with all other preceding studies on this topic) is its cross‐sectional nature of the sample, which cannot establish the causative relationship between occupational exposure and LBP. In the study, self‐reported data were gathered. Certain drawbacks of the report include a lack of workload details and seating structure ergonomics. Although convenience samples were used in the study, which is less transparent than probability samples, we argue that relative to typical convenience samples, homogeneous convenience samples are more appropriate to get a robust result. Furthermore, we were unable to include accurate information on chronic pain or intense pain, or whether the employees consulted a doctor because of the pain or not. Besides, the MET scale may not be sensitive enough for estimating the workload in the selected study population (ie, office workers). Nonetheless, the strength of this study is the use of sample participants from a population who experienced similar work nature, sitting arrangement, and homogeneous work environment. Moreover, the study population has approximately similar education level. Future research should focus on using a longitudinal sample to examine causal relations between a variety of groups of professionals.

## 5 CONCLUSION

We estimated a high prevalence of LBP among bank employees in Dhaka City. Research showed that the LBP is associated with long working hours and several years of employment. In addition, our results add to existing evidence about the adverse effects of obesity on LBP. Through age, the prevalence of LBP appears to have increased, and is more prevalent among female workers. Acceptable physical activity during working hours may be a low‐cost option for the office staff to reduce LBP.

## DISCLOSURE

*Approval of the research protocol: N/A. Informed consent: N/A. Registry and the registration no. of the study/trial: N/A. Animal studies: N/A. Conflict of interest*: The authors declare that they have no conflict of interests.

## AUTHOR CONTRIBUTIONS

MA and AH participated in study conception, design, and coordination of the manuscript. GUA reviewed the manuscript and helped to draft the manuscript. AH also performed statistical analysis and helped to draft the manuscript. All authors approved the final manuscript.

## ETHICAL CONSIDERATION

We explained the aim and objectives of the research to the full‐time bank employees. After having the verbal agreement from the participant, we distributed a written informed consent form and a questionnaire. The ethical committee of the Bangladesh University of Professionals (2019/273) and IRB of North South University (NSU‐IRB‐2019/54) approved the study.

## CONFLICT OF INTEREST

The authors declare that they have no conflict of interests.

## Data Availability

Click here for the data file http://individual.utoronto.ca/ahmed_3/index_files/data/data.html
